# Genome-wide in silico identification of phospholipase D (PLD) gene family from *Corchorus capsularis* and *Corchorus olitorius*: reveals their responses to plant stress

**DOI:** 10.1186/s43141-022-00311-w

**Published:** 2022-02-11

**Authors:** Md. Abu Sadat, Md. Wali Ullah, Md. Sabbir Hossain, Borhan Ahmed, Kazi Khayrul Bashar

**Affiliations:** Basic and Applied Research on Jute Project, Bangladesh Jute Research Institute, Manik Mia Avenue, Dhaka, 1207 Bangladesh

**Keywords:** Phospholipase D, Jute, Plant growth, Gene expression and abiotic stress

## Abstract

**Background:**

Plant grows in nature facing various types of abiotic stresses for their normal growth and development. During abiotic stress, plants evolve different types of mechanisms to survive in a hostile environment. Phospholipase D (PLD) plays important role in the regulation of diverse cellular processes including stress responses in plants. Member of PLD genes are well studied in different model plants; however, their functions in the jute are not clear yet.

**Result:**

In the present study, a total of 12 and 11 PLD genes were identified in the genome of *C*. *capsularis* and *C*. *olitorius*, respectively. The presence of the two conserved HKD motifs in PLD genes except for *CoPLDδ*-*2* in jute suggests their strong lipase activity. Twenty different motifs were found in the identified PLD genes, and PLD-β1, PLD-γ1, and all members of PLD-δ1 of both jute species contained the highest number of motifs. Phylogenetic analysis showed the close evolutionary relationship among the five groups of jute PLD proteins along with the PLD proteins from *Arabidopsis*. Tissue-specific expression pattern of PLDα1-2, PLD-α2, PLDβ1, PLDγ1, and PLDδ1 of two jute species suggested their involvement in plant growth and development. However, the expression pattern of PLDα1-2, PLDα1-3, PLD-α4, PLDδ1, and PLDδ3 indicated their association during waterlogging stress. In addition, PLD-α2, PLDβ1, and PLDδ2 seemed to be involved in drought stress as well as salinity stress.

**Conclusion:**

This genome-wide identification of jute PLD genes from *C*. *capsularis* and *C*. *olitorius* will help to further functional characterization of the PLD genes for developing stress-tolerant jute variety.

## Background

Plants grow in nature where they are constantly facing abiotic and biotic stresses during their growth and development. They need to adjust themselves in nature by adopting the surrounding changes for their survival and it is also crucial to prevent cellular damage [1]. Environmental stresses (abiotic stress) in plants are minimized by several mechanisms including lipid signaling pathway and higher plants respond instantly to overcome the abiotic stresses by the modifications in their cellular process [2, 3]. Phospholipids are secondary messenger in lipid signaling pathway, produced immediately and transiently response in various stresses by the activation of phospholipases or lipid kinases [4].

Phospholipase D (PLD) proteins are member of the lipid signaling pathway that hydrolyzes the bonds of phospholipids for generating the phosphatidic acids (PA) with a free head group choline [5–7]. All eukaryotic PLDs were categorized into three subfamilies (C2-PLD, PX-PH-PLD, and SP-PLD) where C2-PLD subfamily regulates Ca^+2^-dependent while both PX (phox census sequence) and PH (pleckstrin homology) control Ca^+2^-independent activity [8, 9]. In addition, members of the SP-PLD subfamily contain a signal peptide in its N-terminus [10]. Biochemical studies identified an N-terminal phospholipid-binding sequence and two catalytic HKD (HxKxxxxD) motifs for lipase activity in the members of PLDs of all eukaryotic organisms [11].

PLDs has been shown to involve in plant growth and developmental processes under both abiotic and biotic stress [12, 13]. In Arabidopsis, PLDα1 has been shown responsible for PLD activity which stimulated accumulation of ABA and JA in wounding of plants, and also involved in stomatal closure during drought stress [14, 15]. In *Oryza sativa*, PLDβ1 gene was reported to be responsible for seed germination by stimulating ABA signaling pathway as well as protect plant from microbial attack [16]. The first complementary DNA (cDNA) of PLD was cloned in 1994 from castor bean; since then, many PLD proteins were identified from different plants [17]. Recent advances in genome sequencing facility have given an opportunity to dissect the member of PLD genes at the genomic level from any organism. However, the PLD protein family of many organisms including jute has not been studied yet.

Jute is an important natural phloem fiber producing plant with its vegetable properties [18], and contributes the national economy of Bangladesh by earning foreign currency [19, 20]. The quality of jute fiber produced from Bangladesh is incomparable and very much differ from synthetics [21–23]. However, jute production is hampered due to both biotic and abiotic stresses throughout its growing season [24]. Among abiotic stress, water deficiency, water-logging condition, and salinity stress are noticeable for hampering jute yield [25–27]. However, yield loss of jute can be overcome through several way including searching of germplasms and developing new variety through transgenic and genome editing approaches. Therefore, characterization of the genes associated with stress responses in both jute species can be helpful for future breeding programs to improve the traits of jute.

Recent decoding of draft genome sequences of both jute species [28] has opened an opportunity to identify the PLD genes from jute. The present work was focused to identify the PLD genes through bioinformatics analysis to understand their relationship with other reported PLDs leading to stress-tolerant variety development.

## Materials and methods

### Identification of PLD genes from two jute species

For identifying PLD genes from both *Corchorus capsularis* and *Corchorus olitorius* genomes, genomic data of two jute species were downloaded from the National Center for Biotechnology Information (NCBI) database (PRJNA215141 and PRJNA215142). Reference protein sequences of PLD genes from the model plant Arabidopsis and rice along with other plants (cotton, grape, and poplus) were downloaded from the TAIR (http://www.arabidopsis.org), PlantGDB (http://www.plantgdb.org/OsGDB/), Cottongen (https://www.cottongen.org/), and grape database (http://genomes.cribi.unipd.it/ grape/). BLAST tool was carried out for the detection of PLD homologous genes in both jute species. The *E* value threshold was selected at 10^−10^ for this analysis. All putative PLD genes were manually confirmed by the presence of HKD domain responsible for the hydrolysis activity through multiple sequence alignment (https://www.ebi.ac.uk/Tools/msa/clustalo/).

### Analysis of gene structure, domain, motifs, localization, and physiochemical properties

Exon and intron structures of PLD genes of both jute species were determined with the help of online software Gene Structure Display Server 2.0 (GSDS 2.0) (http://gsds.cbi.pku.edu.cn/) through the information of general feature format (GFF). WoLF PSORT, an online-based platform, was used to predict the probable localization of PLD genes in both jute species [29]. Different physical and chemical assessment and related indexes like theoretical isoelectric point (pI), molecular formula, aliphatic index, stability index, and grand average of hydropathicity (GRAVY) were assed with the ProtParam tools (https://web.expasy.org/protparam/) [30, 31].

### Phylogenetic analysis

ClustalW2 software was used to align the selected protein sequences for phylogenetic analysis of PLD proteins from two jute species, Arabidopsis, three cotton species, grape, Medicago, poplus, and peach. With the aligned protein file, a phylogenetic tree was constructed with MEGA X and neighbor-joining method was used for the phylogenetic tree [32, 33] inferred from 1000 bootstrap replicates with other default parameter.

### Expression analysis of PLD genes

Publically available transcriptome data were downloaded from sequence read archive (SRA) and used for the expression pattern analysis of PLD genes of two jute species (*C*. *capsularis* and *C*. *olitorius*). For expression profiling of drought stress and salinity stress condition, data were downloaded from the project PRJNA378897, SRP116874, and SRP116874, respectively [27, 34, 35]. Data of waterlogging stress were collected form the accession number SRP049494, produced by our group earlier. Expression pattern of both jute species were compared by aligning the RNA-Seq reads with reference genomes of jute and then the transcript abundances were measured using the cufflinks v2.2.1 package, visualized by R libraries [36].

## Results

### Identification of PLD genes from two jute species (C. capsularis and C. olitorius) and conservation of HKD domain

**Fig. 1:**
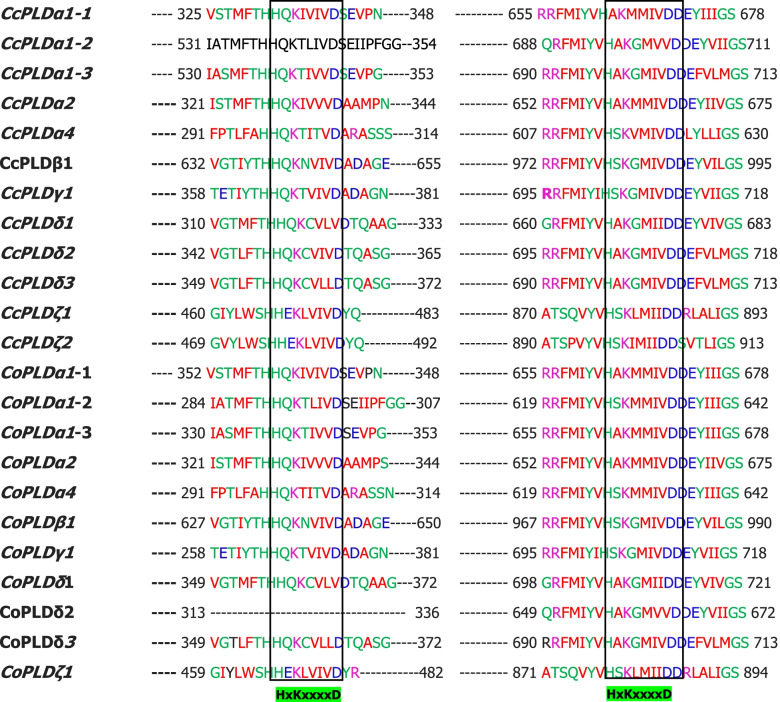
Amino-acid sequence alignment of the PLD genes of *C*. *capsularis* and *C*. *olitorius* containing conserved HKD motifs

To identify the PLD genes from two jute species, protein sequences of PLD genes from Arabidopsis, cotton, grape, poplus, and rice were employed as query, and found 12 and 11 PLD genes in *C*. *capsularis* and *C*. *olitorius*, respectively (Table 1). Based on the nomenclature instruction for plants, Cc and Co symbols were used for *C*. *capsularis* and *C*. *olitorius*, respectively. Depended on the amino acid sequence homology with Arabidopsis, PLD genes of both jute species were divided in five groups namely alpha (α), beta (β), gamma (γ), delta (δ), and zeta (ζ). Both jute species had the similar number of PLD genes in different groups except the zeta (ζ). Analysis also revealed that *C*. *olitorius* genome have one zeta (ζ) containing PLD genes, whereas *C*. *capsularis* have two members of zeta (ζ) containing PLD genes (Table 1). Next, the presence of two HKD domains was analyzed through the amino acid sequence of PLD genes. Analysis of protein sequence found higher conservation of two HKD (HxKxxxxD) domains in all PLD genes of both jute species except *CoPLDδ-2* (Fig. 1). *CoPLDδ*-*2* had only one HKD domain in its protein sequence; however, HKD domains were located far away from each other.

**Table 1:** Basic information of identified PLD genes of *C*. *capsularis* and *C*. *olitorius*

Gene Name	Gene ID	Protein length	Nucleotide length	Number of exon	Number of intron	Subcellular localization
CcPLD-α1-1	CCACVL1_12572	812	2436	3	2	Endoplasmic
CcPLD-α1-2	CCACVL1_23328	821	2463	4	3	Endoplasmic
CcPLD-α1-3	CCACVL1_23327	824	2472	3	2	Endoplasmic
CcPLD-α2	CCACVL1_27885	809	2427	4	3	Endoplasmic
CcPLD-α4	CCACVL1_18644	774	2322	4	3	Cytoplasm
CcPLD-β1	CCACVL1_14191	1129	3387	10	9	Nucleus
CcPLD-γ1	CCACVL1_07714	852	2556	9	8	Cytoplasm
CcPLD-δ1	CCACVL1_27035	818	2454	9	8	Cytoplasm
CcPLD-δ2	CCACVL1_09123	845	2535	10	9	Cytoplasm
CcPLD-δ3	CCACVL1_16893	847	2541	10	9	Cytoplasm
CcPLD-ζ1	CCACVL1_02467	1077	3231	20	19	Cytoplasm
CcPLD-ζ2	CCACVL1_28516	1097	3291	18	17	Cytoplasm
CoPLD-α1-1	COLO4_35115	812	2436	3	2	Endoplasmic
CoPLD-α1-2	COLO4_18852	821	2463	4	3	Endoplasmic
CoPLD-α1-3	COLO4_18851	777	2331	2	1	Endoplasmic
CoPLD-α2	COLO4_12949	809	2427	3	2	Endoplasmic
CoPLD-α4	COLO4_30647	773	2319	4	3	Cytoplasm
CoPLD-β1	COLO4_12643	1124	3372	10	9	Nucleus
CoPLD-γ1	COLO4_23790	852	2556	9	8	Cytoplasm
CoPLD-δ1	COLO4_22499	857	2571	10	9	Cytoplasm
CoPLD-δ2	COLO4_26079	806	2418	10	9	Cytoplasm
CoPLD-δ3	COLO4_11759	847	2541	10	9	Cytoplasm
CoPLD-ζ1	COLO4_03795	1078	3234	20	19	Cytoplasm

### Structure analysis of PLD genes in jute species

**Fig. 2:**
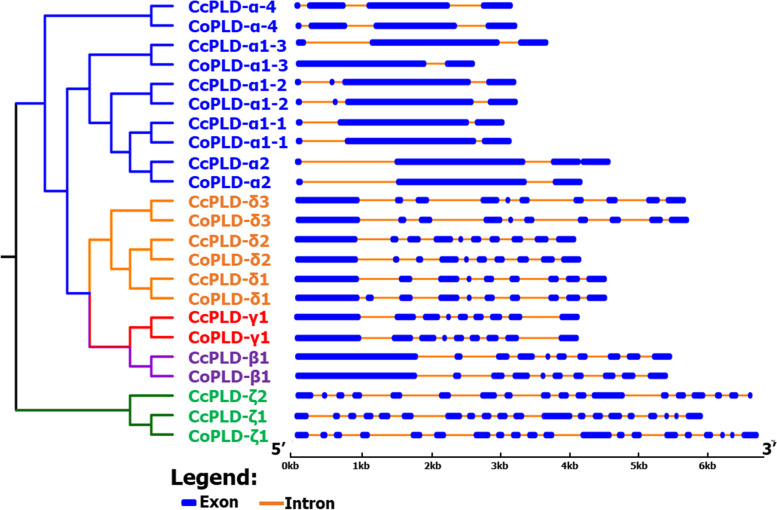
Exon-intron structure of 23 PLD genes of *C*. *capsularis* and *C*. *olitorius*. Exons are shown in blue color and introns are in black lines

**Fig. 3:**
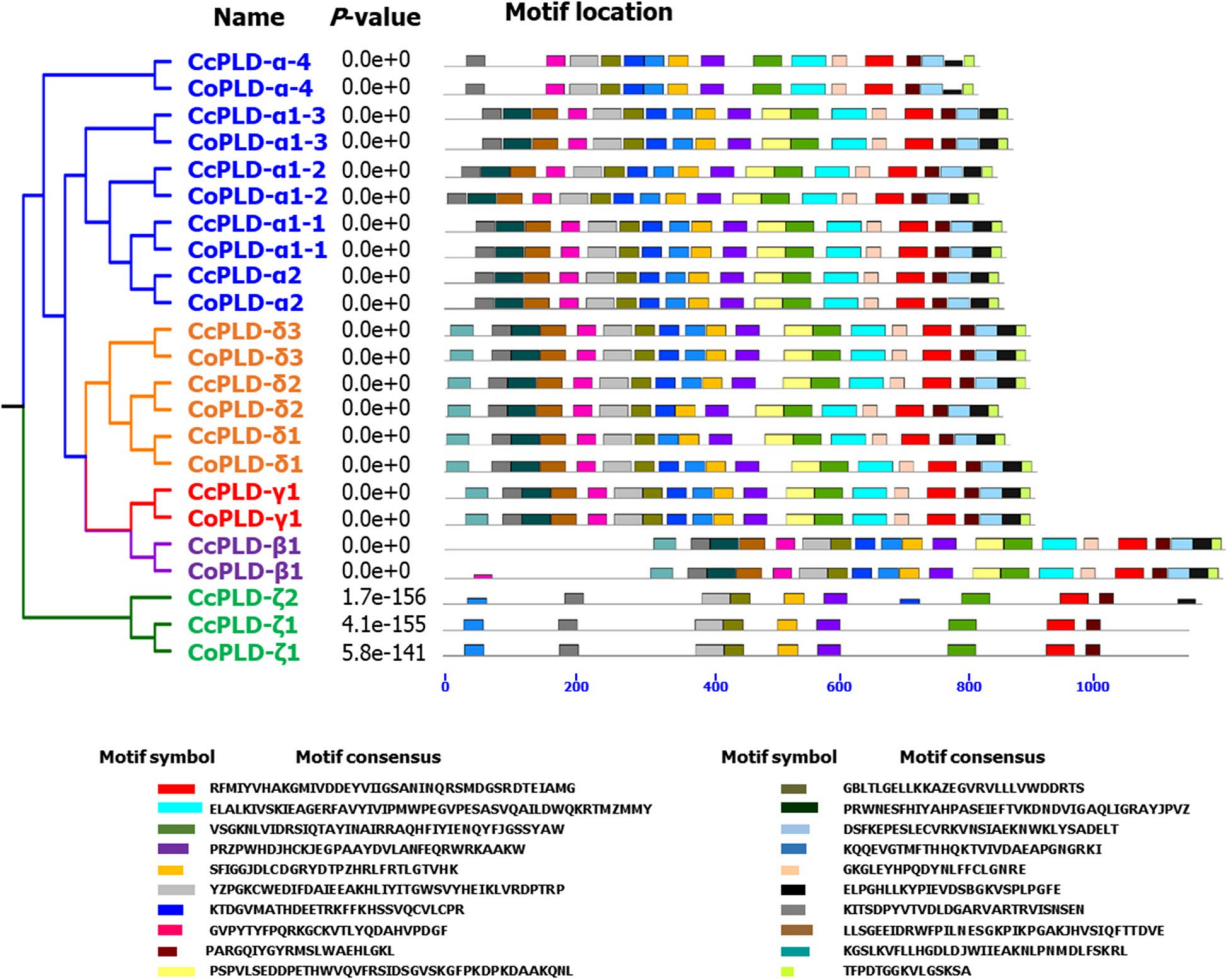
*CcPLD* and *CoPLD* genes and motifs structure. Exons and introns are indicated by boxes and lines, respectively. Motifs are numbered from 1 to 20 and highlighted in different colors. The length of motif for each PLD genes are displayed proportionately. The dendrogram in the left-hand side indicated four distinct phylogenetic groups

Structure analysis of PLD genes found that PLD-ζ1 contained the highest number of exon (20) and intron (19) in both jute species; however, this PLD-ζ1 gene did not have the higher protein length (Table 1 and Fig. 2). The analysis also observed that *CoPLD*-*α1*-*3* from the *C*. *olitorius* had the lower number of exon (2) and intron (1) but not having the lower gene length. In case of gene length, *CcPLD*-*β1* was the longest gene length having 10 exons and 9 introns followed by the *CoPLD*-β1. On the other hand, both *CoPLD*-*α4* and *CcPLD*-*α4* had the lower gene length (Table 1). Subcellular analysis revealed that most of the PLD proteins were found in the cytoplasm (61%) followed by endoplasmic reticulum (35%) (Table 1). However, *CcPLD*-*β1* and *Co PLD*-*β1* solely seemed to be localized in the nucleus. Results from motif analysis revealed that jute PLD genes contained twenty different motifs in their gene sequences (Fig. 3). The analysis detected the PLD-β1, PLD-γ1 of both jute species and *CoPLD*-*δ1* of *C*. *olitorius* contained all motifs in their amino acid sequences; however, motif 8 was duplicated in *CoPLD*-*β1* exceptionally. It was also observed that *PLD*-*α1*-*1*, *PLD*-*α1*-*2*, *PLD*-*α1*-*3*, and PLDα2 had the second highest number of motifs and all members of those genes did not contain the motif number 19 in both jute species. In addition, lowest number of motifs (9 motifs) was found in PLD-ζ1 in both jute species (Fig. 3).

### Physical and molecular characteristics of PLD genes

Different physical and molecular characteristics of PLD proteins were summarized in Table 2. Analysis found variation in theoretical isoelectric point (pl) ranged from 5.4–8.4 and 5.4–8.38 in *C*. *capsularis* and *C*. *olitorius*, respectively which was below 7.0 in most PLD proteins. These results clearly indicated that PLD proteins were slightly acidic to marginally basic. Next, the aliphatic index was measured which help to predict the thermal stability of the protein. The analysis found the higher aliphatic index of PLD proteins ranging from 71 to 87 in both jute species (Table 2). From this result, it can be predicted that PLD proteins are highly thermally stable. Half of the *CcPLD* and *CoPLD* proteins had values less than 40 in the instability index, suggesting their stability in nature. In addition, all the members of PLD protein in both jute species seemed to be hydrophilic as the contained negative value in GRAVY test (Table 2). Molecular weight analysis found the variation in PLD proteins varying from 88 to 122 kDa where higher weight was observed in PLD-β1 and PLD-ζ1 in both jute species. However, the lower molecular weight was found in PLD-α4 in both species and PLD-α1-3 in *C*. *olitorius* species (Table 2).

**Table 2:** List of identified PLD genes along with several physical and chemical properties

Gene Name	Gene ID	Theoretical Pl	Aliphatic index	Instability index	GRAVY	Molecular weight (da)
CcPLD-α1-1	CCACVL1_12572	6.24	84.86	41.36	− 0.371	92,802.87
CcPLD-α1-2	CCACVL1_23328	6.41	84.08	39.33	− 0.396	93,082.90
CcPLD-α1-3	CCACVL1_23327	6.08	85.44	41.29	− 0.459	93,957.79
CcPLD-α2	CCACVL1_27885	5.40	80.27	40.56	− 0.43	91,949.97
CcPLD-α4	CCACVL1_18644	6.89	77.86	40.52	− 0.439	88,663.17
CcPLD-β1	CCACVL1_14191	6.75	71.09	47.7	− 0.514	126,114.87
CcPLD-γ1	CCACVL1_07714	8.64	83.51	38.63	− 0.374	96,031.70
CcPLD-δ1	CCACVL1_27035	6.50	80.81	32.5	− 0.465	93,172.15
CcPLD-δ2	CCACVL1_09123	6.53	79.59	37.47	− 0.344	96,162.48
CcPLD-δ3	CCACVL1_16893	6.52	81.48	35.68	− 0.377	95,999.25
CcPLD-ζ1	CCACVL1_02467	5.91	83.83	43.62	− 0.388	122,332.91
CcPLD-ζ2	CCACVL1_28516	6.49	81.07	50.26	− 0.402	124,644.60
CoPLD-α1-1	COLO4_35115	6.19	84.38	41.54	− 0.388	92,985.85
CoPLD-α1-2	COLO4_18852	6.27	84.19	39.11	− 0.386	92,972.74
CoPLD-α1-3	COLO4_18851	5.95	87.09	43.23	− 0.415	88,328.57
CoPLD-α2	COLO4_12949	5.43	80.26	39.5	− 0.436	91,937.89
CoPLD-α4	COLO4_30647	6.92	77.96	40.5	− 0.419	88,664.18
CoPLD-β1	COLO4_12643	6.92	71.23	48.04	− 0.481	125,576.58
CoPLD-γ1	COLO4_23790	8.38	83.29	36.33	− 0.374	95,903.46
CoPLD-δ1	COLO4_22499	6.64	79.75	32.54	− 0.456	97,286.87
CoPLD-δ2	COLO4_26079	6.38	79.7	37.98	− 0.372	91,822.46
CoPLD-δ3	COLO4_11759	6.50	81.13	35.38	− 0.364	95,826.05
CoPLD-ζ1	COLO4_03795	5.93	82.94	44.04	− 0.406	122,480.92

### Phylogenetic analysis of PLD proteins of two jute species

**Fig. 4:**
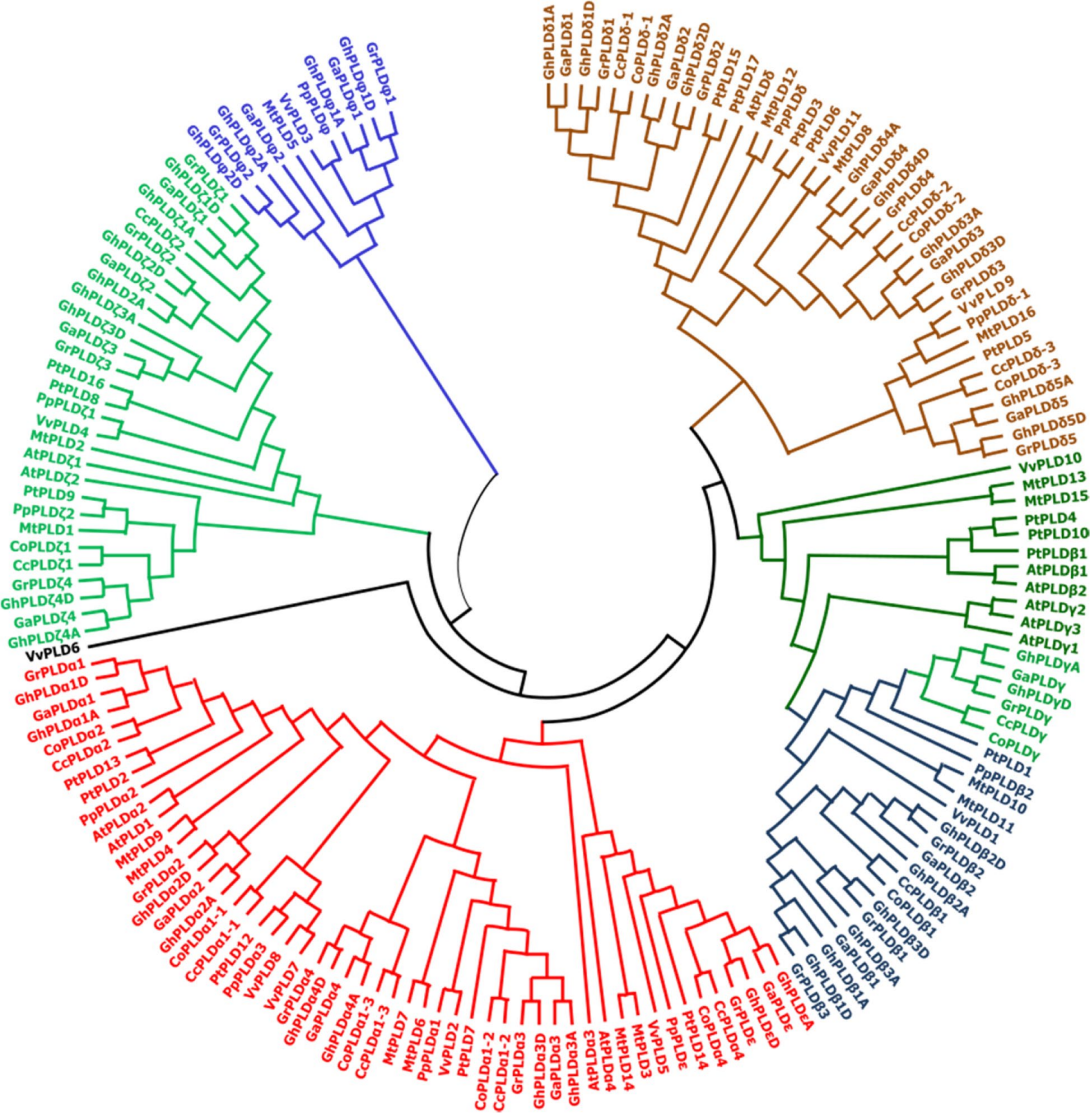
Phylogenetic relationship of PLD proteins among the *C*. *olitorius*, *C*. *capsularis* with different plant species. The phylogeny tree was constructed with MEGA X and neighbor-joining (NJ) method and 1000 replicates bootstraps where Cc, Co, At, Gh, Gr, Ga, Vv, Mt, Pt, and Pp indicates *Corchorus capsularis*, *C*. *olitorius*, *Arabidopsis thaliana*, *Gossypium hirsutum*, *G*. *raimondi*, *G*. *arboretum*, *Vitis vinifera*, *Medicago truncaluta*, *Poplus tremula*, and *Prunus persica*, respectively

By using the amino acid sequence of PLD proteins from two jute species as well as other some plants, a phylogenetic tree was constructed to understand the evolutionary history of the PLD proteins. The phylogenetic tree revealed that 12 CcPLD and 11 CoPLD (five groups) proteins are clustered into five different clades along with the Arabidopsis 12 AtPLD proteins (Fig. 4). Among the different clades, the α type of jute species created the largest clade having 53 members. However, epsilon (ε) isoform of cotton species was found phylogenetically related with alpha (α) isoform of both jute species as well as Arabidopsis. The second largest clade was with the member of PLDδ constituted with 38 members of different plant species (Fig. 4). It was also found that PLDβ and PLDγ of both jute species were phylogenetically closely related as they were under the same clade in the phylogenetic tree (Fig. 4). This might have resulted for the presence of similar motifs in the amino acid sequence and it was observed in the presence of motifs (Fig. 3). From the phylogenetic tree, it was also observed that jute species and Arabidopsis proteins do not contain the isoform phi (φ); however, grape, peach, *Medicago truncaluta*, and cotton species had this PLD isoform (Fig. 4). Members of PLDδ proteins from Arabidopsis and jute species were found to be closely related with PLDβ and PLDγ. However, PLDζ proteins are far away from the rest clades in evolutionary distance, suggesting their sequence characteristics might be varied from the other member of PLD proteins in jute species.

### Expression analysis of jute PLD genes

To investigate the probable functions of PLD genes of both jute species expression results from tissues, drought condition, salinity, and waterlogging conditions were analyzed.

**Fig. 5:**
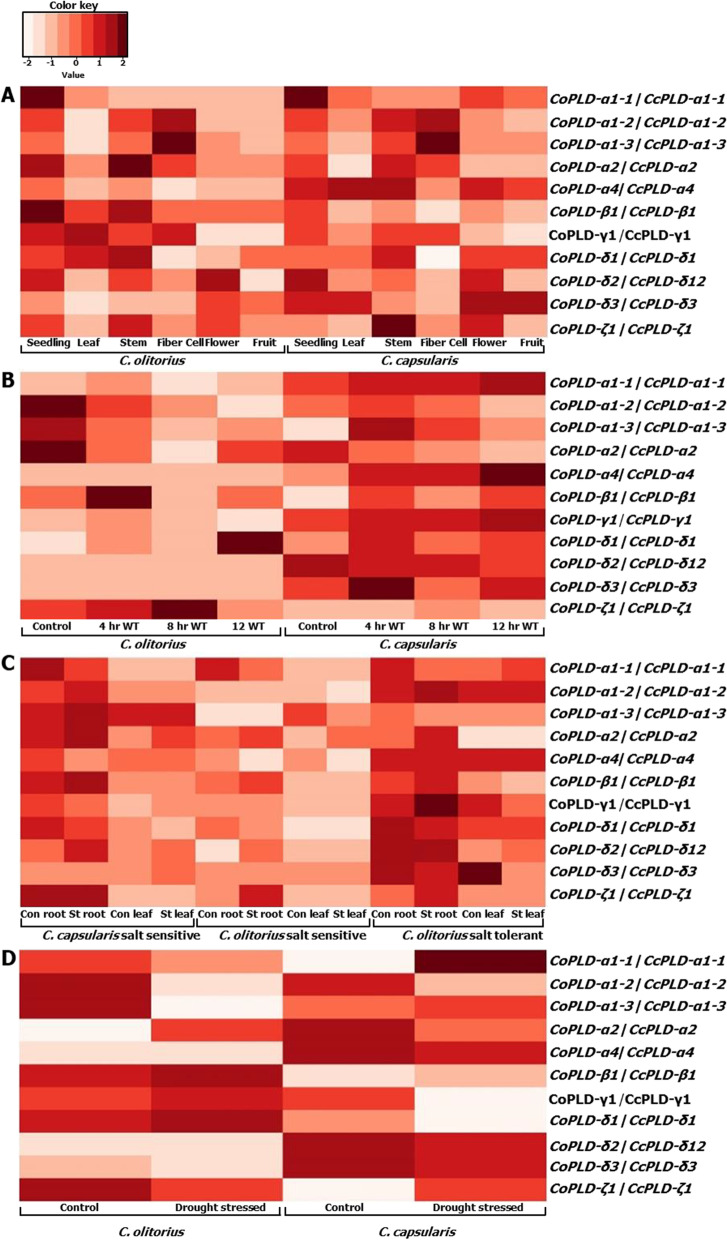
Expression profiling of the member of PLD gene from two jute species (*C*. *capsularis* and *C*. *olitorius*). Tissue-specific gene expression of PLD proteins in various developmental tissues (**A**), in waterlogging stress condition (**B**), in salinity stress condition (**C**), and drought stress condition (**D**). Here, WT, con, and St indicates waterlogging, control, and stress, respectively

In tissue-specific expression profiling, PLDα1-1 highly upregulated only in seedling stage in both jute species compare to the other tissues (Fig. 5A). In addition, *CoPLDβ1* alone highly upregulated in seedling stage than the other PLD genes in two jute species. These results suggested the stage-specific function in jute plant. The analysis also observed higher expression of *CoPLDα1*-*3* and *CcPLDα1*-*3* in fiber cells than the remaining tissues. This result may indicate the specific function of PLDα1-3 during fiber cell formation. Whereas *CoPLD*-α2 and *CcPLD*-*ζ1* showed higher expression patterns in stem cell compared to the other tissues (Fig. 5A). It was also observed that most PLD genes were upregulated in all tissues; however, downregulation was found in leaf and fiber cells. In addition, *CcPLD*-*δ3* showed upregulated expression during the flowering and fruiting stage indicated the importance of this gene for flowering and fruiting for jute.

Under waterlogging condition, expression pattern of three PLD genes of *C*. *capsularis* (*CcPLDα1*-*1*, *CcPLD*-*α4*, and *CcPLDγ1*) gradually increased with the increase of waterlogging periods (Fig. 5B). This result may indicate the involvement of these three PLD genes during waterlogging stress. In case of *C*. *olitorius*, *CoPLD*-*ζ1* showed an increasing expression pattern up to 8 h of waterlogging condition indicating the importance of this gene during early waterlogging condition. Analysis also found that PLD genes of *C*. *capsularis* more or less upregulated under waterlogging stress compare to the PLD genes of *C*. *olitorius*. This result indicated the reasons of being waterlogging tolerance ability of *C*. *capsularis* than the *C*. *olitorius* (Fig. 5B).

In case of *C*. *capsularis* salt*-*sensitive variety, *CcPLDα1*-*2*, *CcPLDα1*-*3*, *CcPLDα2*, *CcPLDβ1*, *CcPLDδ2* were highly unregulated and *CcPLDα1*-*1*, *CcPLDα4*, *CcPLDγ1*, *CcPLDδ1* were downregulated under salt stress condition compared to the control in root (Fig. 5C). Three PLD genes (*CoPLDα2*, *CoPLDβ1*, and *CoPLD-ζ1*) were upregulated in root sample of *C*. *olitorius*. In addition, *CoPLDα1*-*1* was found highly downregulated in root samples in the same jute species. However, expression of PLD genes in salt stressed leaves was not comparable compare to the root sample. Expression pattern of PLD genes in salt-tolerant *C*. *olitorius* jute was also analyzed. Comparative higher and upregulated expression of PLD genes was observed in in root than the samples of leaves (Fig. 5C right panel).

Expression analysis of PLD genes under drought condition revealed that PLDα1-1, PLDα1-3, and PLD-ζ1 was down regulated in *C*. *olitorius*, whereas highly upregulated in *C*. *capsularis* compared to the control condition (Fig. 5D)*.* On the other hand, *PLDβ1*, *PLDγ1*, and *PLDδ1* showed upregulated expression in *C*. *olitorius*; however, comparatively lower and downregulated expression in *C*. *capsularis*. PLDα1-2 showed higher downregulation in both jute species under the same stress condition. Form the above result, it can be predicted that not all but some of the PLD genes might play an important role during drought stress condition.

## Discussion

Plant evolves a number of mechanisms to protect themselves against various environmental stresses for their survival. Phospholipase D (PLD) is a member of phospholipase superfamily which is involved to protect plants from external stresses [37]. PLD gene family of plant play an important role during various stresses such as cold, drought, and salt conditions as well as involved in programmed cell death [16]. However, the member of plant PLDs are more complex than the other organism containing different types of enzymes with noticeable structural, biochemical and regulatory properties.

Number of PLD genes in different plants are not consistent as various reports showed the variation of PLD gene member in different plants [38–42]. Member of PLD gene family has a unique feature of having two HKD (HxKxxxxD) domains far away from each other, however, interact with each other for promoting lipase activity [12]. In this study, a total of 12 and 11 PLD genes were identified from two jute species *C*. *capsularis* and *C*. *olitorius*, respectively, through the bioinformatic analysis (Table 1). The amino acid analysis identified the conservation of two HKD domains in all jute PLD genes except *CoPLDδ2* (Fig. 1). In addition, *C*. *capsularis* genome contained two PLD proteins having zeta isoform (*CcPLDζ1* and *CcPLDζ2*), whereas *C*. *olitorius* genome contains single protein with zeta isoforms (*CoPLDζ1*) (Table 1). Sequence alignment found high sequence similarity between *CcPLDζ1* and *CoPLDζ1* (Data not shown); however, *CcPLDζ2* sequence showed insertion of partial amino acid resulting in more protein length than the *CcPLDζ1* and *CoPLDζ1* (Table 1).

Phylogenetic analysis is one of the amino acid sequence analyses which help to understand not only the relationship of the proteins but also help to predict their evolutionary history [43]. It was also reported that protein functions can be interpreted through the phylogenetic tree and a novel method is required for the genome level understanding [44]. The phylogenetic analysis found four clades for CcPLD and CoPLD proteins along with the Arabidopsis PLD proteins (Fig. 3). Based on sequence similarity, similar results were also reported from the previous studies on rice and Arabidopsis, suggesting the evolution of PLD proteins in different species [45]. It is very likely that PLD proteins in similar clade may have the similar functions and similar prediction was reported in *Solea senegalensis* proteins involved in immune system [46]. In addition, PLDζ proteins of both jute species along with Arabidopsis were found far away from the rest clades in the evolutionary distance indicating their similar evolution with the same function of PLDζ proteins with Arabidopsis [47].

PLD genes are abundant in plant species and significantly involved in salt tolerance [48]. PLDα1 has been previously reported to be highly expressed in different plant organs such as root, stem, leaf, hypocotyl, petal, anther, and fiber in canola [7]. In addition, PLDδ has been reported to play important role in freezing and salt tolerance as well as involved in stomatal closure [49–52]. In silico study on publicly available data, seedling, and stem tissues were more favored for the expression of PLD genes rather than leaf, fiber, flower, and fruit, indicating their involvement in the xylem formation [27, 34, 35]. Tissue-specific expression pattern of *CcPLDα1*-*1*, *CoPLDα1*-*1*, *CcPLDα1*-*3*, and *CoPLDβ1* showed the importance of PLD genes in jute (Fig. 5A). In addition, *CcPLDα1*-*1*, *CcPLD*-*α4*, and *CcPLDγ1* highly upregulated under waterlogging condition (Fig. 5B) which may have an effect on better survivability at excess water conditions. Similarly, several other PLD genes specially *CcPLDα1*-*1*, *CcPLDα1*-*2 CcPLDα2*, and *CcPLDδ2* were upregulated in *C*. *capsularis* than the *C*. *olitorius* under salt and drought stress condition, indicating their important role of PLD genes in *C*. *capsularis* abiotic stress tolerance. Similar hypothesis was also found from one research where *C*. *capsularis* was suggested to be more abiotic tolerance than the *C*. *olitorius* [53].

## Conclusion

In this in silico study, we provided genome-wide identification and analysis of phospholipase D proteins for the first time in natural phloem fiber producing plant jute. A total of 12 and 11 PLD genes were identified from *C*. *capsularis* and *C*. *olitorius*, respectively. Gene structure and phylogenetic analysis showed that jute PLD genes were divided in five groups (α, β, γ, δ, and ζ). Moreover, the position of PLD proteins in the phylogenetic tree suggests a close evolutionary relationship between two jute species. Expression analysis revealed at least five PLD genes (PLDα1-2, PLD-α2, PLDβ1, PLDγ1, and PLDδ1) from both jute species have a significant role in jute growth and development. In addition, the expression pattern of PLDα1-2, PLDα1-3, PLD-α4, PLDδ1, and PLDδ3 suggesting their involvement during waterlogging stress condition. Moreover, only three genes (PLD-α2, PLDβ1, and PLDδ2) seemed to play an important role against drought and salinity stress conditions. These results give an important understanding for developing abiotic stress-resistant jute variety.

## Data Availability

All the protein sequences are available in NCBI data base.

## Abbreviations

PLD: Phospholipase D
NO: Nitric oxide
NCBI: National Center for Biotechnology Information
GSDS: Gene Structure Display Server
BARJ: Basic and Applied Research on Jute Project
GFF: General feature format
MEGA: Molecular Evolutionary Genetics Analysis
cDNA: Complementary DNA
DNA: Deoxyribonucleic acid
